# Risk factors and the natural history of accelerated knee osteoarthritis: a narrative review

**DOI:** 10.1186/s12891-020-03367-2

**Published:** 2020-05-29

**Authors:** Jeffrey B. Driban, Matthew S. Harkey, Mary F. Barbe, Robert J. Ward, James W. MacKay, Julie E. Davis, Bing Lu, Lori Lyn Price, Charles B. Eaton, Grace H. Lo, Timothy E. McAlindon

**Affiliations:** 1 Division of Rheumatology, Allergy & Immunology, Tufts Medical Center, 800 Washington Street, Box #406, Boston, MA 02111 USA; 2 Department of Quantitative Health Sciences, University of Massachusetts Medical School, Worcester, MA USA; 3 Department of Anatomy and Cell Biology, Temple University School of Medicine, 3500 North Broad Street, Philadelphia, PA 19140 USA; 4 Department of Radiology, Tufts Medical Center, 800 Washington Street, Boston, MA 02111 USA; 5 Department of Radiology, University of Cambridge School of Clinical Medicine, Box 218, Level 5, Addenbrooke’s Hospital, Cambridge, CB2 0QQ UK; 6 Department of Radiology, Norwich Medical School, University of East Anglia, Research Park NR4 7U1, Norwich, UK; 7 Milken Institute of Public Health, The George Washington University, 950 New Hampshire Ave NW, Washington, DC 20052 USA; 8 Brigham and Women’s Hospital and Harvard Medical School, 75 Francis Street PBB-B3, Boston, MA 02115 USA; 9 The Institute for Clinical Research and Health Policy Studies, Tufts Medical Center, 800 Washington Street, Box #63, Boston, MA 02111 USA; 10 Tufts Clinical and Translational Science Institute, Tufts University, 800 Washington Street, Box #63, Boston, MA 02111 USA; 11 Center for Primary Care and Prevention, Alpert Medical School of Brown University, 111 Brewster Street, Pawtucket, RI 02860 USA; 12 Medical Care Line and Research Care Line, Houston Health Services Research and Development (HSR&D) Center of Excellence Michael E. DeBakey VAMC, Houston, TX USA; 13 Section of Immunology, Allergy, and Rheumatology, Baylor College of Medicine, Houston, TX. 1 Baylor Plaza, BCM-285, Houston, TX 77030 USA

**Keywords:** Knee, Osteoarthritis, Phenotype, Risk factors, Natural history, Magnetic resonance imaging, Radiography, Meniscus

## Abstract

**Background:**

Osteoarthritis is generally a slowly progressive disorder. However, at least 1 in 7 people with incident knee osteoarthritis develop an abrupt progression to advanced-stage radiographic disease, many within 12 months. We summarize what is known – primarily based on findings from the Osteoarthritis Initiative – about the risk factors and natural history of accelerated knee osteoarthritis (AKOA) – defined as a transition from no radiographic knee osteoarthritis to advanced-stage disease < 4 years – and put these findings in context with typical osteoarthritis (slowly progressing disease), aging, prior case reports/series, and relevant animal models.

**Summary:**

Risk factors in the 2 to 4 years before radiographic manifestation of AKOA (onset) include older age, higher body mass index, altered joint alignment, contralateral osteoarthritis, greater pre-radiographic disease burden (structural, symptoms, and function), or low fasting glucose. One to 2 years before AKOA onset people often exhibit rapid articular cartilage loss, larger bone marrow lesions and effusion-synovitis, more meniscal pathology, slower chair-stand or walking pace, and increased global impact of arthritis than adults with typical knee osteoarthritis. Increased joint symptoms predispose a person to new joint trauma, which for someone who develops AKOA is often characterized by a destabilizing meniscal tear (e.g., radial or root tear). One in 7 people with AKOA onset subsequently receive a knee replacement during a 9-year period. The median time from any increase in radiographic severity to knee replacement is only 2.3 years. Despite some similarities, AKOA is different than other rapidly progressive arthropathies and collapsing these phenomena together or extracting results from one type of osteoarthritis to another should be avoided until further research comparing these types of osteoarthritis is conducted. Animal models that induce meniscal damage in the presence of other risk factors or create an incongruent distribution of loading on joints create an accelerated form of osteoarthritis compared to other models and may offer insights into AKOA.

**Conclusion:**

Accelerated knee osteoarthritis is unique from typical knee osteoarthritis. The incidence of AKOA in the Osteoarthritis Initiative and Chingford Study is substantial. AKOA needs to be taken into account and studied in epidemiologic studies and clinical trials.

## Background

Knee osteoarthritis is typically a slowly progressive disorder. However, approximately 3.4% of adults develop radiographic evidence of accelerated knee osteoarthritis (AKOA) over 4 years [1, 2]. Therefore, at least 1 in 7 cases of incident knee osteoarthritis develop AKOA [1, 2]. We define AKOA as a process characterized by the rapid onset and progression from pre-radiographic disease to advanced-stage radiographic disease in less than 4 years (Kellgren-Lawrence [KL] grades = 0 or 1 to KL = 3 or 4) [1, 3, 4]. For the purpose of this review we will define the “onset” of AKOA as the first visit with radiographic evidence of advanced-stage radiographic disease. Individuals that develop AKOA typically progress from no or doubtful knee osteoarthritis (KL 0 to 1) to definite joint space narrowing and osteophyte (KL = 3) [5]. Two out of 3 adults that develop AKOA will experience this sudden onset and progression (KL 0 or 1 to KL 3 or 4) within 1 year [1, 3–5].

Adults with AKOA represent an important proportion of adults with incident knee osteoarthritis. For example, at least 3 years before radiographic onset adults with incident AKOA have greater pain and disability compared to adults with a typical, gradual onset of knee osteoarthritis (KL 0 to 1, KL 0 to 2, or KL 1 to 2 over 4 years) [5, 6]. Furthermore, while very few people who develop typical knee osteoarthritis receive a knee replacement over 8 years (0.3%), more than 1 in 14 (7%) adults with AKOA undergo a knee arthroplasty within 2.3 years after initial signs of radiographic progression [7]. Including these adults in studies with those who develop typical knee osteoarthritis may yield misleading results in clinical trials and epidemiological studies [3]. Unfortunately, there are no comprehensive reviews to synthesize the risk factors and natural history for AKOA, as well as how AKOA compares with the current paradigm of typical knee osteoarthritis, aging (no radiographic knee osteoarthritis and no KL change over 4 years), and rapidly progressive forms of osteoarthritis. This latter point is particularly relevant because clinicians and researchers often interchange the terms accelerated and rapidly progressive osteoarthritis despite important differences between these disorders. The purpose of this narrative review is to summarize recent evidence from the Osteoarthritis Initiative about the risk factors and natural history of accelerated knee osteoarthritis (AKOA) – defined as a transition between no radiographic knee osteoarthritis to advanced-stage disease within 4 years – and put these new findings in context with typical osteoarthritis, aging, prior case reports/series, and relevant animal models. We acknowledge that the definition of typical knee osteoarthritis may be susceptible to misclassification because of a reliance on subtle changes in KL grades. However, we believe this definition has construct validity because the people with typical knee osteoarthritis differ from those without knee osteoarthritis based on reporting more knee symptoms the year before disease onset [6], reporting a knee injury more often before radiographic changes (a key risk factor for osteoarthritis) [1], and having more meniscal pathology [8].

To achieve our goal, we will start by reviewing the natural history of AKOA and its risk factors. The natural history will be divided into 3 phases: 1) 2 or more years before radiographic osteoarthritis onset, 2) 1 to 2 years before radiographic osteoarthritis onset, and 3) less than 1 year before radiographic onset. Within each phase, we will describe the clinical and structural changes that characterize each phase in comparison to adults with typical knee osteoarthritis or no knee osteoarthritis (representative of age-related changes). After summarizing the natural history of AKOA, we will describe the clinical consequences of AKOA, strategies to classify adults at risk for AKOA, and put AKOA into context with rapidly progressive osteoarthritis and destructive arthropathies.

## Natural history of accelerated knee osteoarthritis and its risk factors

Numerous factors may contribute to AKOA and help people identify who will develop it. Similar to typical knee osteoarthritis, older age and body mass index (BMI) are related to AKOA onset [1, 9, 10]. However, two subgroups are at greater risk for AKOA: 1) individuals < 65 years of age with BMI > 32.5 kg/m^2^ and 2) individuals > 65 years of age that were typically overweight or obese with BMI < 35 kg/m^2^ (only 27% had BMI < 25 kg/m^2^) [10–12]. Greater age and BMI may contribute to the onset of AKOA through pathways related to hyperglycemia and elevated inflammation. However, contrary to this hypothesis, glycated serum protein concentrations, a biomarker of glucose homeostasis, associated with the subsequent onset of typical knee osteoarthritis but not AKOA [13]. There are also no statistical associations between serum concentrations of C-reactive protein or glucose and AKOA onset over the subsequent 4 years to those measurements [13]. It remains unknown if a change in biomarkers of systemic inflammation or glucose homeostasis occurs as people develop pre-radiographic structural changes that antedate radiographic disease onset.

Certain biomechanical factors may also contribute to AKOA. While the presence of static knee malalignment does not significantly differ between individuals who will develop AKOA or typical knee osteoarthritis, greater coronal tibial slope (describing the slope of the tibial plateau relative to a perpendicular line to the long axis of the tibia) is related with a greater odds of incident AKOA but not typical knee osteoarthritis when compared to adults without knee osteoarthritis. However, this relationship was only present among people who had a varus or valgus static malalignment [14]. Hence, a joint may tolerate aberrant coronal tibial slopes in isolation but become susceptible to failure when stressed by combining altered coronal tibial slope with static malalignment (i.e., varus or valgus malalignment).

### 2 or more years before radiographic osteoarthritis onset

#### Clinical

Individuals destined to develop AKOA are more likely to report more knee pain and knee-specific disability, as well as walk slower than adults who will develop typical knee osteoarthritis up to 3 years in advance of disease onset [5].

#### Structural alterations

Magnetic resonance images may reveal why people 2 years in advance of radiographic disease onset are more likely to report greater pain and dysfunction than individuals who develop typical knee osteoarthritis. For example, adults with infrapatellar fat pad signal-intensity alteration or large effusion-synovitis volume, assessed with magnetic resonance imaging, have roughly twice the odds of developing AKOA onset over the subsequent four years [15]. At least 2 years before radiographic onset, people who develop AKOA have greater effusion-synovitis volume compared with those who developed typical knee osteoarthritis [16]. Furthermore, adults who develop AKOA are more likely to have infrapatellar fat pad signal-intensity alteration than those with no knee osteoarthritis [16]. Effusion-synovitis volume and infrapatellar fat pad signal-intensity alteration may be reflective of local inflammation [17] and at least moderately related to knee pain [18–22].

Effusion-synovitis can be both a result of other joint damage or significant stress to a joint [22] or contribute to further aberrant structural changes [23]. Therefore, effusion-synovitis may be a key contributor that perpetuates a vicious cycle defined by joint damage causing effusion-synovitis that leads to worsening pathology and ultimately leads to the accelerated joint decline that is observed in AKOA.

Joint symptoms and effusion-synovitis during this early phase coincide with other structural alterations. For example, a possible sign that the joint experiences abnormal biomechanical loading early in the disease process is that degenerative cruciate ligaments [11, 15], meniscal pathology [15], and thicker medial femoral cartilage (possibly cartilage swelling) [8] are pathologic features on magnetic resonance imaging that may discriminate people who will develop AKOA over the subsequent 2 to 4 years. These pathologic findings may be caused by abnormal arthrokinematics (e.g., laxity) or contribute to abnormal arthrokinematics that lead to local inflammation. In the first scenario, a person may have altered arthrokinematics leading to these pathologic findings because of acquired poor movement patterns or poor neuromuscular control secondary to a large effusion-synovitis volume [24–26]. In the second scenario, the degenerative cruciate ligaments could introduce rotational or antero-posterior knee instability and increased external adduction moments during walking, which contributes to meniscal pathology and large effusion-synovitis volumes [27]. These early risk factors or markers of AKOA may suggest we should explore the benefit of early use of arthrocentesis, anti-inflammatory therapies, or physical rehabilitation focused on neuromuscular control for people at risk for AKOA.

At 2 years prior to radiographic onset, people who developed AKOA have significantly higher odds of having meniscal pathology than controls, especially destabilizing meniscal tears (odds ratio ~ 4.7) [8]. Destabilizing meniscal tears compromise meniscal function and load distribution properties [28, 29] and consist of a radial tear (including a root tear) or complex tear, which almost always includes a radial component [8]. The adults who develop AKOA are also more likely to have meniscal damage in 2 or more regions (66% vs 30%), any medial meniscal pathology (excluding extrusion; 72% vs 39%), and medial meniscal extrusion (20% vs 6%), compared with adults with no knee osteoarthritis over the next 4 years [8].

These early findings highlight that adults who develop AKOA experience greater preradiographic disease burden than their peers who develop typical or no knee osteoarthritis over the next 4 years. It remains unclear why the joint is experiencing excessive disease burden during this early phase. The joint may be susceptible to these early pathologic changes because of a genetic predisposition or developmental susceptibility that leaves their tissues ill-prepared to handle joint loading, or the joint is exposed to excessive overloading with inadequate recovery that leads to increased local inflammation and aberrant tissue changes [30]. While clarifying the etiology of these early changes could yield novel prevention strategies, it is essential to also take steps to identify people without radiographic knee osteoarthritis that report greater joint symptoms and may have magnetic resonance imaging evidence of greater disease burden to help prevent accelerated disease onset and progression.

### 1 to 2 years before radiographic osteoarthritis onset

#### Clinical

Between 2 to 1 year before disease onset people who develop AKOA are more likely to report greater knee pain than earlier assessments [5]. Chair-stand pace and self-reported global impact of arthritis also start to worsen among those who develop AKOA while those with typical knee osteoarthritis stay the same or improve slightly [5]. These worsening symptoms correspond to the start of a dramatic rate of changes within the joint.

#### Structural alterations

During the 2 years before radiographic disease onset, adults who develop AKOA have, on average, a 4.6 times greater increase in effusion-synovitis volume compared with their peers with typical knee osteoarthritis [16]. Additionally, they experience, on average, a 13 times greater increase in bone marrow lesion volume and a greater loss of articular cartilage than adults with typical knee osteoarthritis [8]. Unlike typical knee osteoarthritis, which may be conceptualized with focal cartilage damage, adults who develop AKOA experience diffuse cartilage changes throughout the knee [31]. Overall, these early changes characterize the start of a downward slope towards joint failure. It will be helpful to learn whether an intervention could halt or slow this progression or if they have already passed a point of no return.

### Less than 1 year before radiographic onset

#### Clinical

Within the 12-months before disease onset, people who develop AKOA (31%) or typical knee osteoarthritis (21%) are more likely to report frequent use of medication for pain, aching, or stiffness compared with those with no knee osteoarthritis (10%) [6]. Despite the frequent use of medication to manage symptoms, people who develop AKOA or typical knee osteoarthritis during the next 12 months report greater symptoms in most activities than those without incident knee osteoarthritis. More specifically, people who develop AKOA are more likely to report greater difficulty with lying down, pain with straightening the knee, pain walking, daily knee pain, and frequent knee swelling compared with peers who will develop typical knee osteoarthritis [6].

#### Structural alterations

This peak in prodromal symptoms corresponds to continued worsening throughout the knee; including worsening bone marrow lesions, increasing effusion-synovitis volumes, and more frequent occurrence of infrapatellar fat pad signal-intensity alterations, meniscal damage in 2 or more regions, any medial meniscal pathology or extrusion, or lateral meniscal extrusion than adults with either typical or no knee osteoarthritis [8, 16]. These changes may be secondary to diffuse differences in tissue composition or secondary to joint instability.

### A new knee injury – especially within 1 year before radiographic onset

**Fig. 1:**
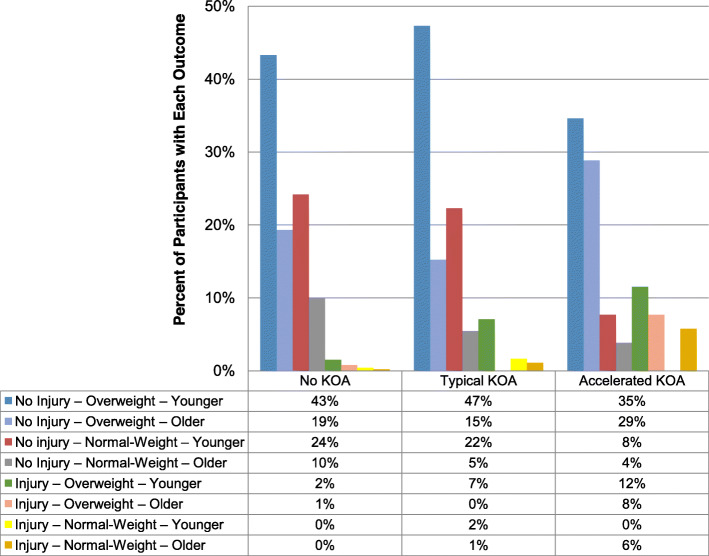
The distribution people across groups that develop accelerated, typical, or no knee osteoarthritis **(KOA).** The distribution of subsets of people in the Osteoarthritis Initiative defined by age, body mass index, and injury across groups that develop accelerated, typical, or no knee osteoarthritis (KOA) [10]. The percentages in the data table are based on the percent of people with accelerated, typical, or no KOA that are in each subset (each column adds to ~ 100% due to rounding) [10]

If a susceptible knee has large effusion-synovitis or degenerative cruciate ligaments that contribute to altered lower extremity biomechanics, then it is no surprise that these adults also report more knee injuries than their peers. Specifically, a single new injury may be a critical event that leads to joint failure or characterizes the onset of AKOA [1, 9, 32]. A history of a knee injury several years before the onset of AKOA is unrelated to disease onset [1, 9]. However, an injury in the year or two before incident knee osteoarthritis is more common among adults that develop AKOA or typical knee osteoarthritis than those who do not develop knee osteoarthritis [1]. Furthermore, a knee injury may be especially likely to lead to AKOA among normal weight and older individuals (65 years or older) or overweight-obese individuals (Fig. 1) [10]. Among people with incident AKOA, 26% were older or overweight/obese and reported a new injury in the year before developing disease onset. In contrast, only 8 and 3% of people with incident typical knee osteoarthritis or no knee osteoarthritis were older or overweight/obese and reported a new injury (Fig. 1) [10]. Hence, a new knee injury to a joint already susceptible because of other risk factors may increase the risk of an accelerated rate of joint failure.

The interaction between other risk factors and a knee injury is consistent with evidence from the destabilizing medial meniscal (DMM) mouse model. The DMM model is a surgical model that relies on destabilizing the medial meniscus to mimic human clinical meniscal injury, especially a destabilizing meniscal tear [33, 34]. The natural history of disease progression after DMM is similar to spontaneous onset [33, 35]; however, greater age [36, 37] and high-fat diets [38] are risk factors for accelerated disease onset after DMM. Furthermore, while a sex-age interaction is not reported among studies of AKOA, there is evidence that this interaction may be relevant in the DMM model. With a DMM model, orchiectomized male mice (lower testosterone) had less severe osteoarthritis than other males while ovariectomized female mice (lower sex hormones) had greater disease severity than other females. The interaction between injury, sex, sex hormones, and age may explain why simplistic analyses of sex differences in AKOA are null and, therefore, a more nuanced study may be needed [34].

We must be cautious about relying solely on self-reported injury because adults who develop AKOA may fail to perceive a traumatic event as a knee injury [32]. For example, among people who develop AKOA that failed to report an injury, almost 40% had distinct structural changes on magnetic resonance images, typically incident medial meniscal pathology [32]. Hence, it became critical to understand the key structures that experienced trauma around the time of disease onset. Ultimately, destabilizing meniscal tears (i.e., radial tears, root tears, complex tears) and trauma to the subchondral bone characterized the trauma that led to or characterized the onset of AKOA instead of typical knee osteoarthritis [8, 32, 39, 40].

By the time people present with AKOA, they are > 7 times more likely to have a destabilizing meniscal tear than those without AKOA [8]. Furthermore, over 90% of adults with AKOA have meniscal damage in 2 or more regions, 85% have medial meniscal pathology, and 77% of individuals with AKOA have meniscal extrusion [8].

It is paramount that we identify people prior to this devasting trauma and strive to prevent trauma, which is likely the tipping point from which a joint is unable to recover and ultimately leads to a quick onset of joint failure. Collectively, this evidence supports a call that if a patient is referred to physical therapy for knee pain they may benefit from establishing a goal to prevent a new joint injury that could either be the catalyst for joint failure or define a pivotal moment in the process of joint failure.

### Summary of natural history

**Fig. 2:**
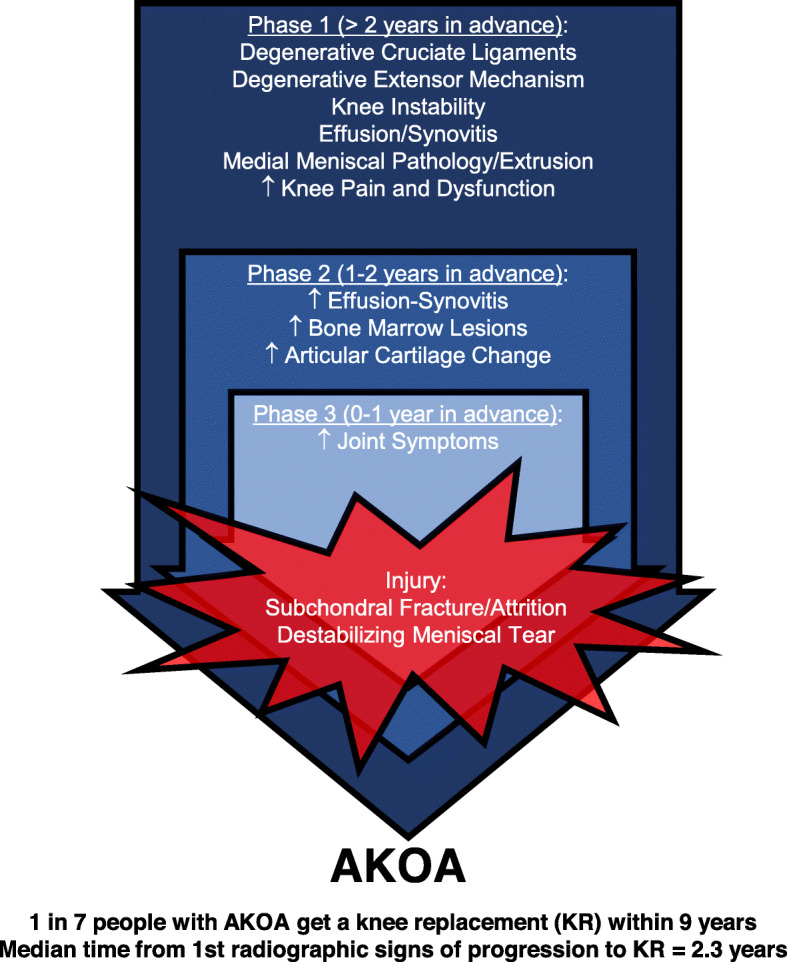
Phases of the Natural History of Accelerated Knee Osteoarthritis

In summary, the natural history of incident AKOA can be conceptualized as three phases (Fig. 2). Starting at least 2 years in advance of radiographic onset, adults with AKOA may experience greater effusion-synovitis, thicker articular cartilage, diffuse meniscal pathology (including destabilizing meniscal tears), and degenerative changes in the cruciate ligaments and extensor mechanism. Hence, during this early phase, a joint is already experiencing greater preradiographic disease burden than their peers, which may explain why they report more prodromal symptoms than those who develop typical knee osteoarthritis. The subsequent 12-month phase is defined by a dramatic rate of worsening in effusion-synovitis, bone marrow lesions, and articular cartilage (Fig. 2).

Within 12 months before radiographic onset, adults with AKOA report more joint symptoms, frequent use of pain medication, frequent knee swelling, and daily knee pain compared with those who develop typical knee osteoarthritis. The greater knee pain could contribute to a new knee injury, which is often characterized by a destabilizing meniscal tear. The joint trauma may be a triggering event in a joint with an impaired ability to heal, which ultimately leads to joint failure. The compromised ability to heal in response to an injury may complement evidence from young adult rats performing a high-repetition, high-force reaching and lever pulling task [30, 41]. This model may suggest that the damage-repair theory may be relevant to trabecular changes [42] and that damage accumulates in the bone if the loading is so high that self-repair mechanisms cannot keep pace with the level of damage or overloading-induced bone resorption. When rats perform the high-repetition, high-force task for 18 weeks, they exhibit several catabolic indices in trabeculae in their distal radial metaphyses, including decreased trabecular bone volume, increased woven bone, osteoclast numbers, microcracks and osteocyte apoptosis, compared to control rats. The loss of trabecular bone volume enhances brittleness and increases fracture risk [43]. These catabolic bone changes with high demand tasks are also consistent with the fatigue-failure theory for musculoskeletal disorder injuries [44]. Hence, overloading a knee joint beyond its capacity to repair may contribute to the accumulation of bone damage, which could explain why adults with AKOA develop larger bone marrow lesions and are more likely to develop attrition or subchondral insufficiency fractures than their peers [8, 39].

## Clinical consequences of accelerated knee osteoarthritis

At an individual level, we’ve described how adults with AKOA experience greater symptoms and functional impairments starting up to 3 years prior to radiographic disease onset. This complements findings that over the first 8 years of the Osteoarthritis Initiative, people who develop AKOA are more likely to report receiving arthroscopic knee surgery, intra-articular injections (i.e., hyaluronic acid, corticosteroids), and nonsteroidal anti-inflammatory drugs (over the counter or prescription) than people who develop typical or no knee osteoarthritis [45]. Even prior to the onset of radiographic evidence of AKOA, people who will develop AKOA are more likely to use certain prescription analgesics (e.g., acetaminophen, celecoxib, aspirin) and receive arthroscopic knee surgery than those who will develop typical knee osteoarthritis [45].

The greater personal burden of AKOA is further highlighted by the high rate of knee replacements. While most people who started the Osteoarthritis Initiative without radiographic knee osteoarthritis did not receive a knee replacement over the first 9 years of the study (≤ 1%), almost 1 in 7 knees with AKOA received a knee replacement during that period [7]. Furthermore, the median time from any increase in radiographic severity (change in KL grade) to knee replacement was 2.3 years (range 0.3 to 7.3 years) compared with the few knees with typical osteoarthritis that received a knee replacement (3.0 years; range 1.7 to 4.2 years) [7]. Adults with AKOA experience significant disease burden and have only a short window of opportunity to intervene, with 1 in 14 knees receiving a knee replacement in less than 2.3 years after the first evidence of radiographic progression.

Another concern is that our perception of osteoarthritis may be biased by our failure to account for people with AKOA. Within the Osteoarthritis Initiative, AKOA accounts for more than 1 in 5 cases of incident knee osteoarthritis [1]. Hence, factors that have a strong relationship with AKOA, but not typical knee osteoarthritis, may still appear associated with knee osteoarthritis overall. For example, excluding people with incident AKOA from analyses that defined incident knee osteoarthritis as at least a 2-point increase in radiographic severity led to smaller effect estimates (Cohen’s d [46]) when comparing cases of incident disease to controls: knee pain over time (d = 0.41 for everyone, d = 0.14 after excluding AKOA) or recent knee injury (OR = 5.4 for everyone, OR = 4.0 after excluding AKOA) [3]. It is essential that we critically assess our current conceptual model of knee osteoarthritis by accounting for differences among phenotypes or clinically relevant subsets (e.g., AKOA). Furthermore, we need to consider the consequences of recruiting people with AKOA into clinical trials. Separate trials may be necessary for AKOA and other types of knee osteoarthritis given AKOA has a distinct natural history defined by the onset of a destabilizing meniscal tear in a joint already susceptible to failure. A critical question is whether people with AKOA will respond differently to therapeutic interventions when compared to those with typical osteoarthritis.

## Classifying adults at risk for accelerated knee osteoarthritis

To develop prevention strategies and clinical trials for adults at risk for AKOA, it is crucial to identify who will likely develop AKOA. Riddle and colleagues developed a prediction rule to estimate the chance of incident AKOA over the subsequent 5 years and found that the presence of possible knee osteoarthritis (KL = 1), contralateral knee osteoarthritis, greater BMI, and joint symptoms (total Western Ontario and McMaster Universities Osteoarthritis Index [WOMAC] score) increased the chance of someone developing AKOA [4]. While this prediction rule provided valuable insights into identifying people at risk for AKOA, it inadequately addressed the complex interactions among the risk factors, and it lacked MR-based structural features that may help classify individuals at risk for AKOA. Hence, we used classification and regression tree (CART) analyses, which can 1) identify the most statistically important factors and the associated cut points to most efficiently differentiate groups for classifying individuals, and 2) account for complex interactions that may optimize the ability to classify individuals at risk for AKOA. Furthermore, we used the CART analyses to explore if MR-based structural findings could help classify people at high risk for AKOA (54 people developed AKOA and 108 sex-matched Osteoarthritis Initiative participants). There is consistent evidence that age, fasting glucose concentrations, and static knee alignment were statistically important clinical factors to consider when classifying people at risk for incident AKOA [11, 12]. Effusion-synovitis volume and cruciate ligament degeneration may also be statistically important for classifying people at risk for AKOA over the subsequent 4 years [11]; however, adding data from magnetic resonance imaging (e.g., quantitative effusion-synovitis, cartilage damage, bone marrow lesions; semiquantitative assessments of menisci, tendons, ligaments) failed to improve the ability to classify adults who develop AKOA over the models with only clinical measures (Table 1). Furthermore, the models failed to explain the majority of the variance that contribute to someone being classified as incident AKOA or not. Hence, we are either missing a major factor that contributes to classifying people with AKOA or there are numerous factors that contribute a little to the classification. Going forward, it will be important to better understand the earliest phases of AKOA to develop strategies to identify people at risk for or with early-stage AKOA.

**Table 1:** Performance of Classification Rules for People who Will Develop AKOA Over the Subsequent 4 Years

	Clinical Model^**a**^	Clinical + MRI Model^**b**^
Specificity	0.82	0.90
Sensitivity	0.70	0.62
Explained Variance (%)	41%	39%

Note: AKOA = accelerated knee osteoarthritis, MRI = magnetic resonance imaging

^a^The final clinical classification tree included age, body mass index, serum glucose concentrations, femorotibial alignment angle, serum glycated serum protein concentrations, WOMAC knee-related function score, and coronal tibial slope angle [11]

^b^The final clinical+MRI classification tree included body mass index, serum glucose concentrations, effusion-synovitis volume, presence of cruciate ligament degeneration, and coronal tibial slope angle [11]

## Accelerated knee osteoarthritis within the context of rapidly progressive osteoarthritis and destructive arthropathies

Numerous terms have been used during the last 60 years in the medical literature to describe a dramatic change in joint health; including rapidly progressive disease, rapidly destructive disease, accelerated degeneration, massive osteolysis, rapid chondrolysis, among others. The heterogeneity in nomenclature reflects uncertainty about how to classify rapidly destructive forms of osteoarthritis and their underlying mechanisms.

It is informative to reassess the existing literature and consider how various definitions may impact the reported epidemiology of rapidly progressive or accelerated osteoarthritis. For example, in Table 2, we offer several definitions of accelerated or rapidly progressive osteoarthritis and how the incidence using these definitions vary from 0 to 22% within the Osteoarthritis Initiative.

**Table 2:** Frequency of Accelerated or Rapid KOA Using Previously Reported Definitions Among Adults without Radiographic KOA at OAI Baseline

Term	Radiographic Change	Time Frame	Incidence in OAI
Accelerated OA [1, 3, 4]	KL 0 or 1 to KL 3 or 4	4 or 5 years	7.2%
Rapid Radiographic Change [47]	KL 0 or 1 then KL change > 2	4 or 5 years	12.4%
Fast JSW Loss [48]	> 0.25 mm medial JSW change	1 year	15.5 to 22.1%
Rapid Progressors [49]	> 1.05 mm medial JSW change	1 year	0.4 to 0.8%
Rapid Destructive Arthrosis [50]	> 2 mm JSW change or 50% narrowing	1 year	0%
Rapid Progressive OA Type 1 [46, 47]	> 2.00 mm JSW change	1 year	0%
Rapid Progressive OA Type 2 [46, 47]	abnormal bone loss or destruction	short period of time	0%

Note: *KOA*  knee osteoarthritis, *OAI*  Osteoarthritis Initiative, *OA*  osteoarthritis, *JSW*  joint space width

Recently, two classifications for rapidly progressive osteoarthritis have been adopted to characterize the rapid joint destruction observed in clinical trials for anti-nerve growth factor (NGF) agents [51, 52]. Rapid progressive osteoarthritis type 1 is similar to an early definition provided by Lequesne [50] that requires more than 2 mm of loss in joint space width in less than one year. Rapid progressive osteoarthritis type 2 is defined by abnormal bone loss or destruction (e.g., osteolysis) [51, 52]. Within the context of the anti-NGF clinical trials, these types of rapid progressive osteoarthritis usually (but not always) manifest in a joint in which osteoarthritis was already present [53]. In contrast, research focused on AKOA has focused on incident disease [4, 40, 54]. While these differences may be consequent on the differences in research design and setting, it remains unclear whether AKOA and rapid progressive osteoarthritis type 1 are separate or overlapping entities. A clear distinction between AKOA and rapid progressive osteoarthritis is that adults with AKOA rarely experience the rate of joint space loss nor the dramatic bone destruction observed with rapid progressive osteoarthritis. For example, no one with AKOA in the Osteoarthritis Initiative experienced more than 2 mm of loss in joint space width in less than one year nor abnormal bone loss or destruction. Furthermore, rapid progressive osteoarthritis is often described in knees, hips, or shoulders [46, 50, 53, 55, 56]. However, accelerated osteoarthritis is primarily observed in the knee and, to a lesser extent, the hand. Therefore, it is unclear how rapid progressive osteoarthritis at large ball-and-socket joints relates to findings at the knee.

Despite differences between AKOA and rapid progressive osteoarthritis, some interesting similarities may be worth exploring further. Both are more common among older adults and may be antedated by certain analgesic medications [45]. Furthermore, people who develop AKOA or rapid progressive osteoarthritis have early evidence of cartilage degradation followed by an extreme rate of articular cartilage loss [8, 31, 57] and early evidence of inflammation [11, 15, 16, 57]. Finally, 12% of knees that develop AKOA experience attrition or subchondral fractures [8] and 35% of people who develop accelerated hand osteoarthritis develop new central erosions in other hand joints [58]. While people with accelerated osteoarthritis never experience the dramatic bone destruction or collapse seen with some types of rapid progressive osteoarthritis it may be beneficial to further explore the role of bone changes in each type of osteoarthritis.

Until we reach greater clarity about the pathogeneses of these subsets of osteoarthritis, it may be inappropriate to conflate them in clinical practice or research studies. Furthermore, we need to be cautious about collapsing these phenomena together or extracting results from one type of osteoarthritis to another. Further studying the similarities and differences between AKOA and other forms of rapidly progressive osteoarthritis may yield new insights into these subsets of osteoarthritis.

## Conclusions

Accelerated osteoarthritis is distinct from typical osteoarthritis. The incidence of AKOA is likely greater than commonly perceived and could have a profound impact on epidemiologic studies and clinical trials. There is an urgent need to consider this subset of osteoarthritis when performing clinical research and to create standard nomenclature for the array of arthropathies that may be related but distinct from AKOA.

## Data Availability

The datasets analyzed during current study are available in the OAI repository, https://nda.nih.gov/oai

## Abbreviations

AKOA: Accelerated knee osteoarthritis
BMI: Body mass index
DMM: Destabilizing medial meniscal
JSW: Joint space width
KL: Kellgren-Lawrence
KOA: Knee osteoarthritis
MRI: Magnetic resonance imaging
NGF: Nerve growth factor
OA: Osteoarthritis
OAI: Osteoarthritis Initiative
OR: Odds ratio
